# Causal Inference in Non-Allergic Asthma Associated with Obesity: Application of the Bradford Hill Viewpoints to a Complex Epidemiological Association

**DOI:** 10.3390/epidemiologia7040107

**Published:** 2026-08-06

**Authors:** José J. Leija-Martinez, Eduardo Ensaldo-Carrasco, Fausto Sánchez-Muñoz, Blanca E. Del-Río-Navarro, Nayely Reyes-Noriega, Fengyang Huang

**Affiliations:** 1 Hospital Regional de Alta Especialidad “Dr. Ignacio Morones Prieto”, IMSS-Bienestar, Av. Venustiano Carranza No. 2395, San Luis Potosi C.P. 78290, San Luis Potosi, Mexico; 2 Centro de Investigaciones en Ergonomía, Centro Universitario de Arte, Arquitectura y Diseño (CUAAD), Universidad de Guadalajara, Calzada Independencia Norte No. 5075, Guadalajara 44250, Jalisco, Mexico; eduardo.ensaldo@gmail.com; 3 Departamento de Fisiología, Instituto Nacional de Cardiología Ignacio Chávez, Mexico City 14080, Mexico; fausto22@yahoo.com; 4 Laboratorio de Investigación en Alergia e Inmunología, Hospital Infantil de México Federico Gómez, Dr. Márquez 162, Cuauhtemoc, Mexico City 06720, Mexico; blancadelrionavarro@gmail.com (B.E.D.-R.-N.); naye.rey.nor@gmail.com (N.R.-N.); 5 Laboratorio de Investigación en Obesidad y Asma, Hospital Infantil de México Federico Gómez, Mexico City 06720, Mexico; huangfengyang@gmail.com

**Keywords:** non-type 2 asthma, neutrophilic airway inflammation, Th17 cells, RORC, interleukin-17A, Mendelian randomisation, body mass index, DNA methylation, adipose tissue inflammation, glucagon-like peptide-1 receptor agonists

## Abstract

**Background/Objective:** Obesity has been linked to a distinct non-allergic, late-onset, neutrophilic asthma phenotype for more than two decades, yet whether obesity should be regarded as a cause of that phenotype rather than a coexisting comorbidity remains unresolved because randomised allocation to obesity is neither feasible nor ethical. This review asks whether the evidence accumulated between 1995 and 2026 supports a causal interpretation. **Methods:** A structured narrative synthesis was undertaken. PubMed/MEDLINE, Scopus and Web of Science were searched from January 1995 to June 2026 (the synthesis emphasises the 2020–2026 evidence while drawing on the full window for landmark studies) for cohort, cross-sectional, Mendelian randomisation (MR), interventional, mechanistic and pharmaco-epidemiological studies; landmark earlier work was retained where it remains the primary source for a given viewpoint. The retrieved evidence was mapped onto Sir Austin Bradford Hill’s nine viewpoints (1965) and triangulated using E-values for unmeasured confounding, GRADE, directed acyclic graphs and MR. **Results:** All nine viewpoints were satisfied to varying degrees. Strength: effect estimates 1.4–6.8, E-values 4.8–13.1, MR summary risk ratio 1.05 per 1 kg/m^2^. Consistency: replication across four continents, both sexes and multiple study designs. Temporality: prospective cohort and life-course MR evidence. Biological gradient: body mass index dose–response for TNFα, IL-17A and Th17 frequency. Plausibility: a twelve-layer architecture from adipose dysfunction to bronchial epithelium. Coherence, experimental evidence (bariatric surgery, lifestyle weight loss, GLP-1 receptor agonists, murine and in vitro models) and analogy were also supported; specificity was met at the phenotype level. **Conclusions:** The cumulative evidence strongly supports a causal contribution of obesity to the development of the late-onset, non-allergic asthma phenotype while falling short of definitive proof (GRADE: moderate certainty), with implications for primary prevention, endotype-targeted therapy and longitudinal research. Phenotype-stratified MR remains the principal missing element of the causal argument.

## 1. Introduction

Causal inference in observational epidemiology is more challenging than in experimental research because investigators cannot manipulate exposures. This challenge is particularly pronounced for multifactorial chronic diseases, where the proposed cause, such as obesity, represents a complex and slowly evolving phenotype. Randomised allocation to obesity is neither feasible nor ethical. As a result, the evidence base comprises cohort, case–control, cross-sectional, mechanistic, and quasi-experimental studies of varying quality. Therefore, a structured heuristic is required to assess whether associational evidence supports a causal interpretation and to identify gaps in the evidence.

The most widely cited heuristic for causal inference is the framework established by Sir Austin Bradford Hill in his 1965 Presidential Address to the Section of Occupational Medicine of the Royal Society of Medicine [1]. Hill proposed nine “aspects of associations”—strength, consistency, specificity, temporality, biological gradient, plausibility, coherence, experiment, and analogy—as viewpoints rather than as a checklist. Recent methodological scholarship has reinforced this interpretation [2,3].

Asthma in the setting of obesity is a paradigmatic test case for causal inference in observational respiratory epidemiology. Holguin and colleagues [4] partitioned the obese asthmatic population into an early-onset, allergic phenotype, in which obesity is a comorbidity of pre-existing type 2 (T2) asthma and a late-onset, non-allergic phenotype, in which obesity precedes asthma and is associated with neutrophilic, steroid-resistant, female-predominant, clinically severe disease. The non-allergic phenotype is reflected in the 2025 update of the Global Initiative for Asthma (GINA) strategy report [5], which lists obesity as a modifiable risk factor for exacerbations and recommends weight loss as an adjunctive treatment.

Before the framework is applied, the phenotype under discussion is defined operationally because several partially overlapping terms recur in this literature and are not interchangeable. Throughout this review, the target phenotype is the non-allergic, obesity-associated asthma phenotype described by Holguin et al. [4]. The other terms in use describe different levels of the same disease rather than synonyms: “late-onset” refers to the clinical timing of onset; “non-T2” (or “non-Th2”) to the inflammatory endotype; “neutrophilic” to the predominant airway cell; “steroid-resistant” to the treatment response; and “obesity-related” or “obesity-associated” to the presumed aetiology. In the typical patient, these features co-occur, but they are conceptually distinct and are not invariably concordant in a given individual. Table 1 sets out the operational definition across the clinical, endotypic and biomarker levels and contrasts it with the allergic (type 2) phenotype. Where the evidence discussed pertains to asthma in general rather than to this specific phenotype, this is stated explicitly.

A second decade of evidence has materially changed the inferential landscape. Bidirectional and life-course Mendelian randomisation (MR) analyses now provide genetic-instrumental triangulation [11,12,13,14,15]; updated cohort studies and meta-analyses have refined strength and biological gradient [16,17,18,19]; epigenome-wide association studies have extended methylation findings beyond their original cohorts [20,21]; the immunological architecture has been complemented by single-cell transcriptomic, NLRP3-inflammasome and lipidomic data [22,23]; and pharmaco-epidemiological studies of glucagon-like peptide-1 (GLP-1) receptor agonists, with an ongoing dedicated randomised controlled trial, have introduced a quasi-experimental modifier of the proposed exposure [24,25,26]. The Mexican context is particularly relevant: the National Health and Nutrition Survey 2022 reported overweight in 38.3% and obesity in 36.9% of Mexican adults, with abdominal obesity in 81.0% [27], and most paediatric asthma in Latin America is non-atopic [28].

In parallel, the clinical and mechanistic literature on this phenotype has been consolidated. Liu et al. [6] reviewed the molecular basis of non-T2 severe asthma and the treatment strategies it implies; Althoff et al. [29] provided a state-of-the-art account of obesity-related asthma linking pathobiology to existing and emerging therapies; Listyoko et al. [30] synthesised the evidence on obesity and airway remodelling; Shailesh et al. [9] catalogued the non-T2 mechanisms operating in obese asthmatic individuals; and Jiang et al. [31] reviewed immune dysregulation in obesity across organ systems. Methodological scholarship on Hill’s framework has advanced alongside: Lesko and Fox [2] traced the evolution of the viewpoints and their influence on epidemiological methods, and Shimonovich et al. [3] proposed a transparent process-tracing approach to their application within systematic reviews. The convergence of these two strands—substantive consolidation of the phenotype on the one hand, methodological refinement of the framework on the other—makes a formal re-examination of the causal question both feasible and timely.

The objective of this review is to determine whether the evidence accumulated between 2020 and 2026 supports a causal interpretation of the association between obesity and the late-onset, non-allergic, neutrophilic asthma phenotype rather than an interpretation in which obesity is merely a coexisting comorbidity. Four specific aims follow from this objective. The first is to apply each of Hill’s nine viewpoints systematically to the contemporary evidence base, stating for every viewpoint the evidence that supports it, the evidence that opposes it, and the residual uncertainty. The second is to quantify the robustness of the principal effect estimates to unmeasured confounding using E-values [32] and to triangulate observational estimates against genetic-instrumental estimates obtained by Mendelian randomisation [11,12,13,14,15,33]. The third is to set out the mechanistic chain linking adipose tissue dysfunction to neutrophilic airway inflammation in sufficient detail for biological plausibility to be assessed on its merits rather than asserted. The fourth is to identify explicitly the methodological gaps that currently prevent the causal claim from reaching the highest evidentiary tier. Throughout, Hill’s framework is applied in the context of its 1964 U.S. Surgeon General’s Report antecedent [34] and of the modern toolkit of GRADE [35], counterfactual inference [36,37], directed acyclic graphs (DAGs) [36] and Mendelian randomisation [33]. The authors’ own translational research on the TNFα–IL-17A–RORC axis and its epigenetic regulation in Mexican adolescents [7,8,38] is incorporated into the evidence base.

### Scope and Approach

This work is a structured (narrative) review underpinned by a systematic literature search. It is not a systematic review in the PRISMA sense: no quantitative meta-analysis was performed, and no single risk-of-bias instrument was applied across the whole corpus for reasons set out below. Three bibliographic databases were searched—PubMed/MEDLINE, Scopus and the Web of Science Core Collection—from 1 January 1995 to 1 June 2026, with the search restricted to the English and Spanish languages. The start date of 1995 was chosen because the modern characterisation of the obesity-associated asthma phenotype and of its inflammatory mechanisms dates from the mid-1990s; the window was extended to June 2026 to capture the most recent evidence. Consistent with the aim of the review, the synthesis emphasises the new evidence published between 2020 and 2026, while the full search window ensures that the landmark earlier studies on which several viewpoints depend are captured. The complete search strings for all three databases are given in the Supplementary Material. A small number of foundational methodological and historical sources that predate the search window—among them being Hill’s 1965 address [1], the 1964 U.S. Surgeon General’s Report [34], and the methodological papers on GRADE [35], the E-value [32] and Mendelian randomisation [33]—were included as citations outside the date-limited search.

The search strategy was adapted from the authors’ registered systematic review protocol on immune mechanisms in childhood-obesity-related asthma (PROSPERO CRD42023472442). Two deliberate broadenings were made so that the strategy would serve a Bradford Hill causal appraisal rather than a paediatric meta-analysis. First, the paediatric restriction was removed: adult and paediatric evidence are both eligible here because the causal question spans the life course and much of the strongest cohort and Mendelian randomisation evidence is in adults. Second, the human-only restriction was removed: animal and in vitro/translational studies are eligible because Hill’s viewpoints of experiment and analogy depend on murine and mechanistic models (for example, the diet-induced obesity model in which IL-17A elevation precedes airway hyperresponsiveness [39]), which a human-only filter would have excluded. In addition, terms for Mendelian randomisation, longitudinal and life-course cohorts, quasi-experimental weight loss and GLP-1 receptor agonist studies, and epigenome-wide association studies were added to the design block so that every category of evidence relevant to the nine viewpoints could be retrieved.

Eligible study types were therefore human observational studies (prospective and retrospective cohort, case–control and cross-sectional), Mendelian randomisation studies, interventional and quasi-experimental studies (bariatric surgery, structured weight loss, and GLP-1 receptor agonist pharmaco-epidemiology), epigenetic and epigenome-wide association studies, and animal or in vitro mechanistic studies; integrative reviews were retained as background and contextual sources. Records were screened for their capacity to inform one or more of Hill’s viewpoints, and the reference lists of the retrieved reviews were hand-searched (snowballing). Because the resulting corpus spans designs as heterogeneous as murine mechanistic experiments and genome-wide Mendelian randomisation, no single risk-of-bias instrument is applicable to all of it; the Newcastle–Ottawa scale used in the parallel systematic-review protocol, for instance, does not apply to animal or Mendelian randomisation studies. Study quality was therefore appraised in two complementary ways: the design features of the principal observational studies (prospective versus cross-sectional design, adjustment set, and sample size) are reported in the text and in Table 2, and the robustness of the leading observational estimates to unmeasured confounding is quantified with E-values and triangulated against genetic-instrumental estimates from Mendelian randomisation. This quantitative triangulation is, for a design-heterogeneous body of evidence, a more informative substitute than a single scoring instrument that no such body could share. Finally, because part of the mechanistic argument draws on the authors’ own translational work [7,8,38], findings generated by the authors’ group are identified as such throughout and are distinguished from independent replication wherever the latter exists. This review is distinct in scope and method from the authors’ parallel paediatric systematic review (CRD42023472442), which addresses a narrower question with formal meta-analysis.

## 2. The Bradford Hill Viewpoints in Their Original Form

Hill delivered his 1965 address in the context of the smoking–lung cancer debate, presenting his nine viewpoints not as criteria but as “aspects of that association which we should especially consider before deciding that the most likely interpretation of it is causation” [1]. He cautioned that none could “bring indisputable evidence for or against the cause-and-effect hypothesis” and that none, except temporality, could be “required as a sine qua non.” The subsequent popularisation of these as “Hill’s criteria” has narrowed the original intent. In this review, the viewpoints are employed as Hill intended: as structured perspectives that organise heterogeneous evidence into a coherent causal argument, triangulated with quantitative tools unavailable in 1965, including the E-value for unmeasured confounding [32], Mendelian randomisation [11,12,13,14,15,33], DAGs [36], and GRADE [35].

## 3. Application of the Nine Viewpoints to Non-Allergic Asthma Associated with Obesity

**Figure 1:**
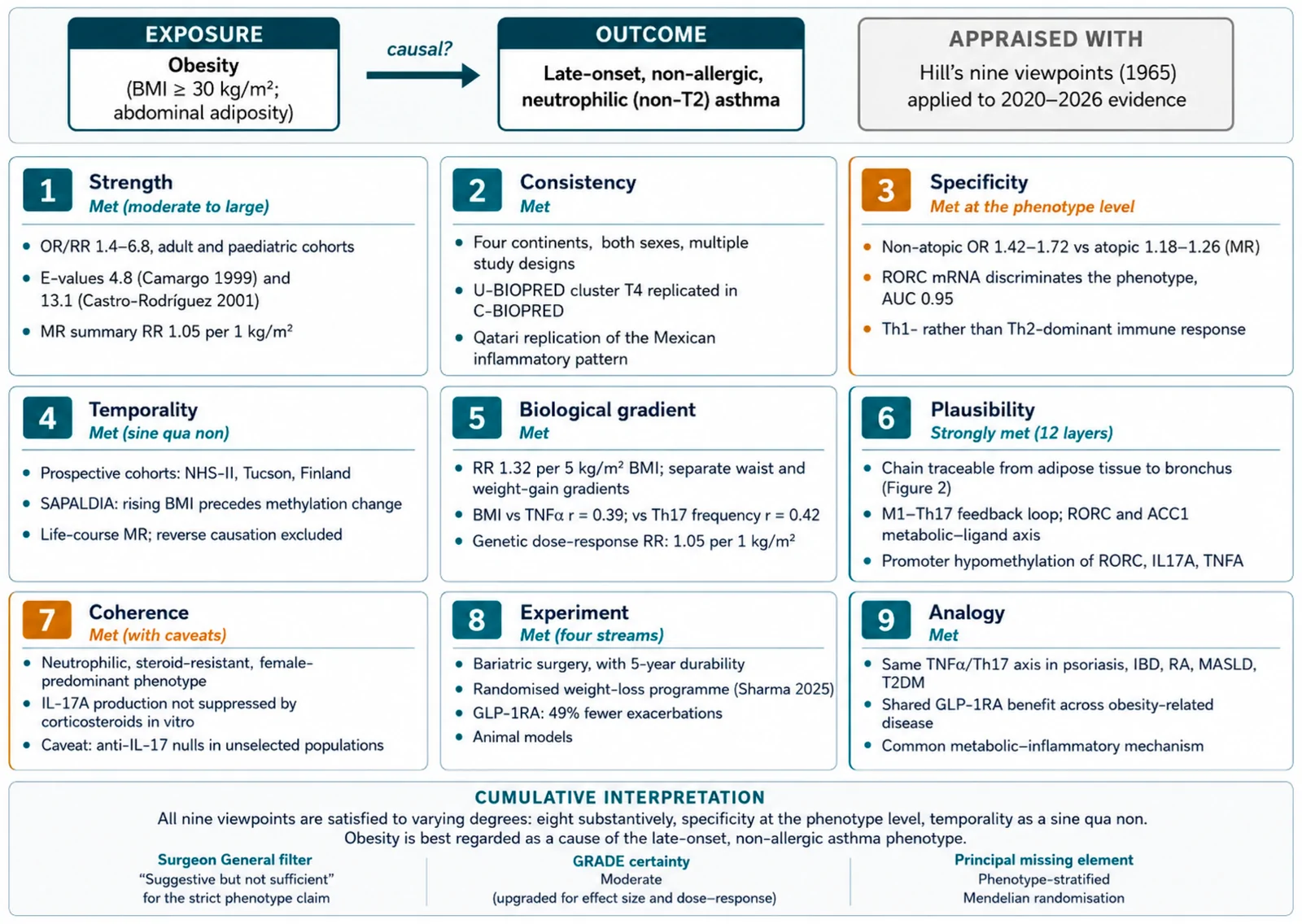
Schematic summary of the nine Bradford Hill viewpoints [1] applied to the association between obesity and late-onset, non-allergic, neutrophilic (non-T2) asthma. For each viewpoint, the panel states the status assigned in this review, the principal quantitative evidence and the corresponding reference numbers; the accent bar indicates the strength of support (dark: strongly met or met as a sine qua non; medium: met; amber: met at the phenotype level or met with caveats). The evidence shown in each panel is supported by the following references: (1) *Strength*—effect estimates from adult and paediatric cohorts and meta-analyses [16,17,18,19,40,41,42,43,44,45], E-values of 4.8 and 13.1 calculated for the estimates of Camargo et al. (1999) and Castro-Rodríguez et al. (2001) [32,40,42], and the Mendelian randomisation summary estimate [15]; (2) *Consistency*—replication of the U-BIOPRED T4 cluster in C-BIOPRED [46,47] and replication of the Mexican inflammatory pattern in a Qatari paediatric cohort [48]; (3) *Specificity*—phenotype-restricted Mendelian randomisation estimates for non-atopic versus atopic asthma [11], discrimination of the phenotype by RORC mRNA expression [7], and the Th1- rather than Th2-dominant immune response [49]; (4) *Temporality*—prospective cohorts (NHS-II, Tucson, Finland) [17,40,42], the SAPALDIA observation that rising BMI precedes methylation change [50], and life-course Mendelian randomisation excluding reverse causation [11,12]; (5) *Biological gradient*—BMI, waist and weight-gain gradients [16], correlations of BMI with TNFα [51] and with Th17 frequency [52], and the genetic dose–response relationship [15]; (6) *Plausibility*—the twelve-layer mechanistic chain from adipose tissue to bronchus shown in Figure 2, comprising the M1–Th17 feedback loop and the RORC–ACC1 metabolic–ligand axis [53,54,55,56,57,58] and promoter hypomethylation of *RORC*, *IL17A* and *TNFA* [8,38,59]; (7) *Coherence*—the neutrophilic, steroid-resistant, female-predominant phenotype [4,6,9], corticosteroid-refractory IL-17A production in vitro [10], and the null results of anti-IL-17 therapy in unselected populations [60]; (8) *Experiment*—bariatric surgery with five-year durability [61,62], a randomised weight-loss programme [63], the reduction in exacerbations with GLP-1RA [26], and diet-induced obesity models [39]; (9) *Analogy*—the shared TNFα/Th17 axis across psoriasis, IBD, RA, MASLD and T2DM [31] and the shared benefit of GLP-1RA across obesity-related disease [24,25,26]. The footer gives the cumulative interpretation, the corresponding Surgeon General evidentiary tier [34], the GRADE certainty rating [35], and the principal outstanding methodological gap. Detailed evidence for each viewpoint is presented in Section 3.1, Section 3.2, Section 3.3, Section 3.4, Section 3.5, Section 3.6, Section 3.7, Section 3.8 and Section 3.9 and summarised with supporting data in Table 3. Abbreviations: AUC, area under the receiver operating characteristic curve; BMI, body mass index; GLP-1RA, glucagon-like peptide-1 receptor agonist; GRADE, Grading of Recommendations Assessment, Development and Evaluation; IBD, inflammatory bowel disease; MASLD, metabolic-dysfunction-associated steatotic liver disease; MR, Mendelian randomisation; OR, odds ratio; RA, rheumatoid arthritis; RORC, retinoid-related orphan receptor C; RR, relative risk; T2DM, type 2 diabetes mellitus.

Each of the nine viewpoints is examined in turn below. Figure 1 summarises the appraisal schematically, giving for each viewpoint its status, the principal quantitative evidence and the corresponding references; the sections that follow set out the underlying evidence in full, and Table 3 restates the summary in tabular form.

### 3.1. Strength of Association

In the prospective Nurses’ Health Study II, Carlos A. Camargo Jr. et al. [40] reported an odds ratio (OR) of 2.7 (95% CI 2.3–3.1) for incident asthma among women who became obese. The classic 2007 meta-analysis by David A. Beuther and Elliot R. Sutherland [41] (seven cohorts, 333,102 participants; OR 1.92, 95% CI 1.43–2.59) has since been superseded by the 2023 systematic review and meta-analysis by Parasuaraman et al. [16], which included more than sixteen cohorts and demonstrated a monotonic dose–response relationship between body mass index (BMI) and adult asthma incidence, with separate gradients for waist circumference and weight gain. A total of sixteen studies (63,952 cases and 1,161,169 participants) were included in the quantitative synthesis. The pooled relative risk (RR) was 1.32 (95% CI 1.21–1.44; I^2^ = 94.6%, p_heterogeneity < 0.0001; n = 13) per 5 kg/m^2^ increase in BMI, 1.26 (95% CI 1.09–1.46; I^2^ = 88.6%, p_heterogeneity < 0.0001; n = 5) per 10 cm increase in waist circumference, and 1.33 (95% CI 1.22–1.44; I^2^ = 62.3%, p_heterogeneity = 0.05; n = 4) per 10 kg increase in weight gain. Overall, there was a clear dose–response relationship between increasing adiposity and asthma risk.

Two additional large cohort studies published in 2024 further support these findings. Vartiainen et al. [17], in a cohort of 59,668 Finnish adults, demonstrated that obesity (BMI ≥ 30 kg/m^2^) increased asthma risk in both sexes independently of smoking and physical activity. During follow-up, 4612 (14%) women and 2578 (9.3%) men developed asthma. Compared with the reference BMI category (<24.9 kg/m^2^), obesity was associated with hazard ratios (HRs) of 1.57 (95% CI 1.44–1.71) in women and 1.63 (95% CI 1.44–1.83) in men.

Similarly, Bloodworth et al. [18], in a cohort of 90,081 US adults, disentangled the contributions of adiposity and components of metabolic syndrome, demonstrating that both independently predicted incident asthma. Among the 90,081 eligible participants, 836 incident asthma cases (0.93%) were identified. Diabetes mellitus at baseline, in the absence of other metabolic syndrome components, was associated with an increased risk of asthma (adjusted HR 1.85, 95% CI 1.27–2.71; *p* = 0.0002). Independent of other metabolic syndrome components, overweight and obesity were associated with a 10-year attributable asthma risk of 15.4%.

In paediatric populations, effect sizes appear even larger. The Tucson Children’s Respiratory Study by José A. Castro-Rodríguez et al. [42] demonstrated that girls aged 6–13 years who became overweight or obese had an OR of 6.8 (95% CI 2.4–19.4) for asthma at age 13.

The Taiwanese paediatric cohort study by Chen et al. [19] evaluated the causal association between obesity and childhood asthma using a Mendelian randomisation (MR) design. A total of 7069 children aged 12 years from the Taiwan Children’s Health Study were included. Cross-sectional logistic regression, one-sample MR, summary-level MR sensitivity analyses, and prospective survival analyses were used to investigate causal pathways. In the prospective survival analysis, obesity was associated with the highest risk of incident asthma per 1-interquartile-range increase (HR 1.28, 95% CI 1.05–1.56).

Three paediatric meta-analyses further support these findings [43,44,45]. Chen et al. [43] analysed three cohorts involving 14,083 children and reported an RR of 2.02 (95% CI 1.16–3.50). Egan et al. [44] analysed two cohorts involving 15,688 children and found an RR of 1.50 (95% CI 1.22–1.83). Likewise, Deng et al. [45], in eight cohorts comprising 73,252 children, reported an OR of 1.40 (95% CI 1.29–1.52).

Collectively, these effect sizes are best characterised as moderate at the population level and large within specific subgroups. They exceed the threshold at which residual confounding by a single unmeasured covariate would provide a parsimonious alternative explanation. From a quantitative perspective, the E-value [32] required to fully explain away the OR of 2.7 reported by Camargo et al. [40] is approximately 4.8. In contrast, the E-value corresponding to the OR of 6.8 reported by Castro-Rodríguez et al. [42] is 13.1. Consequently, an unmeasured confounder would need to be associated with both obesity and incident asthma, with relative risks of at least 4.8 and 13.1, respectively, even after adjustment for measured covariates, to nullify the observed associations. Plausible confounders, such as physical activity, dietary patterns, socioeconomic position, and indoor allergen exposure, rarely show associations of this magnitude with both the exposure and the outcome simultaneously. Applying the same calculation to the lower confidence limits, E-values of 4.0 and 4.2, respectively, would be required for an unmeasured confounder to shift these confidence intervals to include the null.

Finally, the Mendelian randomisation meta-analysis by Mikkelsen et al. [15] provides a complementary causal estimate of approximately 1.05 (95% CI 1.03–1.07) per 1 kg/m^2^ increase in BMI, with the genetic-instrumental signal being substantially stronger for non-atopic and adult-onset moderate-to-severe asthma phenotypes.

Table 2 summarises the design, population, exposure metric and effect estimate of each of the studies discussed in this section.

**Table 2:** Studies informing the strength of the association between adiposity and incident asthma, in chronological order of publication.

Study (Reference)	Design	Population	Exposure Metric	Effect Estimate (95% CI)	Principal Finding
Camargo et al., 1999 [40]	Prospective cohort (Nurses’ Health Study II)	Adult women, USA	Incident obesity during follow-up	OR 2.70 (2.30–3.10)	Women who became obese had a markedly raised risk of incident adult-onset asthma; E-value 4.8.
Castro-Rodríguez et al., 2001 [42]	Prospective birth cohort (Tucson Children’s Respiratory Study)	Children aged 6–13 years, USA	Becoming overweight or obese during the school years	OR 6.80 (2.40–19.40)	Effect confined to girls; the largest effect size in the literature; E-value 13.1.
Beuther & Sutherland, 2007 [41]	Meta-analysis of 7 prospective cohorts	333,102 adults	Overweight or obesity vs. normal weight	OR 1.92 (1.43–2.59)	Established the association at meta-analytic level; superseded by Parasuaraman et al. [16].
Chen et al., 2013 [43]	Meta-analysis of 3 cohorts	14,083 children	Childhood overweight or obesity	RR 2.02 (1.16–3.50)	Sex difference in the prediction of incident childhood asthma.
Egan et al., 2013 [44]	Meta-analysis of 2 cohorts	15,688 children	Childhood body mass index	RR 1.50 (1.22–1.83)	Higher childhood BMI precedes physician-diagnosed asthma.
Deng et al., 2019 [45]	Meta-analysis of 8 cohorts	73,252 children	Overweight or obesity	OR 1.40 (1.29–1.52)	Consistent but smaller effect in childhood than in adolescence and adulthood.
Chen et al., 2022 [19]	One-sample and summary-level Mendelian randomisation with prospective survival analysis (Taiwan Children’s Health Study)	7069 children aged 12 years	Obesity, per 1-interquartile-range increase	HR 1.28 (1.05–1.56)	Genetic-instrumental support for a causal pathway in childhood.
Mikkelsen et al., 2022 [15]	Systematic review and meta-analysis of Mendelian randomisation studies	Population-based genetic consortia	Genetically predicted BMI, per 1 kg/m^2^	RR 1.05 (1.03–1.07)	Signal substantially stronger for non-atopic and adult-onset moderate-to-severe asthma.
Parasuaraman et al., 2023 [16]	Systematic review and dose–response meta-analysis of 16 cohorts	63,952 cases among 1,161,169 participants	BMI, per 5 kg/m^2^; waist circumference, per 10 cm; weight gain, per 10 kg	RR 1.32 (1.21–1.44); RR 1.26 (1.09–1.46); RR 1.33 (1.22–1.44)	Monotonic dose–response for all three adiposity metrics; I^2^ 62.3–94.6%.
Vartiainen et al., 2024 [17]	Prospective cohort	59,668 middle-aged adults, Finland	BMI ≥ 30 vs. < 24.9 kg/m^2^	HR 1.57 (1.44–1.71) women; HR 1.63 (1.44–1.83) men	Independent of smoking and physical activity; 4612 (14%) female and 2578 (9.3%) male incident cases.
Bloodworth et al., 2024 [18]	Real-world retrospective cohort	90,081 adults, USA	Overweight/obesity and components of metabolic syndrome (diabetes mellitus at baseline)	10-year attributable risk 15.4%. HR 1.85 (1.27–2.71)	Adiposity and metabolic components independently predict incident asthma; 836 incident cases (0.93%).

Abbreviations: BMI, body mass index; CI, confidence interval; HR, hazard ratio; OR, odds ratio; RR, relative risk. E-values were calculated as E = RR + √[RR × (RR − 1)] [32].

### 3.2. Consistency

The association has been demonstrated in adult cohorts in the United States [18,40,41], Northern Europe [17], and in paediatric cohorts in Asia [43], the United States [42], and Latin America [28]. Cross-sectional inflammatory studies in Turkey, Korea, Germany, Italy, Poland, China, the Gulf region, and Mexico report concordant findings on Th17 frequency and elevated IL-17A levels in obese subjects [7,8,38,48]. Latin American consistency is particularly relevant. Cooper and colleagues [28], within the Social Change, Asthma and Allergy in Latin America (SCAALA) programme, established that most paediatric asthma in Latin America is non-atopic, with risk strongly associated with diet, obesity, and psychosocial stress. The Mexican Global Asthma Network Phase I data [64] report wheezing in the past 12 months of 10.2% among children and 11.6% among adolescents, compared with the contemporary obesity prevalence reported in ENSANUT 2022 [27].

A 2025 Qatari cross-sectional study by Shailesh et al. [48] replicated the inflammatory pattern in 364 children. Asthma was independently associated with elevated levels of IL-17A, IL-22, IL-33, and TNF-α. Obesity moderated rather than amplified these elevations through statistically significant interactions. The Qatari replication of earlier Mexican findings in a culturally and dietetically distinct population is a strong signal of consistency. Cluster-analytical replication is also informative. The U-BIOPRED Cluster T4 [46]—predominantly obese female patients with uncontrolled severe asthma—has been replicated in the Chinese C-BIOPRED severe asthma study [47]. Sex-specificity (stronger effects in females) [15,19,42] refines rather than undermines consistency. Earlier Mexican null findings in preschool and school-age children [65,66] fit within the broader pattern that the obesity–asthma association is weaker in early childhood than in adolescence and adulthood, consistent with the life-course MR finding that adult adiposity drives the adult-onset phenotype.

### 3.3. Specificity

Specificity is the weakest of Hill’s viewpoints; Hill himself acknowledged that one-cause-one-effect relationships are rare in chronic disease. Modern reformulations reinterpret specificity at the effect level rather than at the exposure level. Read this way, the obesity–asthma association is remarkably specific. Holguin et al. [4] used cluster analysis on Severe Asthma Research Programme data to identify the late-onset, non-allergic phenotype, characterised by Th17 polarisation, elevated IL-17A and TNFα, neutrophilic airway inflammation, and a distinctive transcriptional signature involving the NLRP3–IL1B–IL17 axis [9,22]. The Nyambuya meta-analysis confirmed a Th1-dominant rather than Th2-dominant immune response in this phenotype [49].

Mendelian randomisation evidence considerably sharpens the specificity argument. Sun et al. [11] reported odds ratios per 1-SD (4.1 kg/m^2^) increase in BMI of 1.42–1.72 for non-atopic asthma versus 1.18–1.26 for atopic asthma, with reverse causation excluded through bidirectional MR. Wang et al. [13] showed that whole-body and trunk fat mass causally increase the risk of asthma. Urquijo et al. [12] showed that adult adiposity drives the adult-onset phenotype more strongly than childhood adiposity does. Together, these MR analyses provide phenotype-level specificity on a genetic-instrumental basis that observational evidence alone cannot deliver.

Data from the authors’ research align with this phenotype-level specificity. In a cross-sectional study of 102 Mexican adolescents, stratified into healthy controls, allergic asthma without obesity, obesity without asthma, and non-allergic asthma with obesity, serum TNFα, serum IL-17A, and the messenger RNA expression of TNFA, IL-17A, and RORC in peripheral blood leukocytes were specifically elevated in the non-allergic-asthma-with-obesity group. The methylation profile of the *RORC* promoter was specifically reduced [7,8]. *RORC* messenger RNA expression discriminated non-allergic asthma among obese adolescents, with an area under the receiver operating characteristic curve of 0.95 (95% CI 0.86–0.99) [7], a discriminative performance approaching diagnostic test thresholds. At the molecular level, the non-allergic-with-obesity phenotype was distinguishable from the other three groups in ways that the allergic-asthma-without-obesity group was not.

### 3.4. Temporality

Temporality is the only Hill viewpoint that is genuinely a sine qua non for causation. For obesity and asthma, the temporal direction is supported by several independent prospective cohorts. In the Nurses’ Health Study II [40], women who became obese during follow-up had a substantially elevated risk of subsequently developing asthma. In the Tucson Children’s Respiratory Study [42], girls who became overweight before age 11 had a significantly elevated incidence of asthma-like symptoms at age 13. The Vartiainen 2024 Finnish cohort [17] adds Northern European replication. Most pertinent to the late-onset, non-allergic phenotype, the Swiss SAPALDIA cohort study [50] followed adults for 10 years and found that those with subsequent increases in BMI developed adult-onset non-atopic asthma. This was accompanied by detectable epigenetic changes in inflammatory pathways, including reduced methylation of CpG sites in the NLRP3–IL1B–IL17 axis. This temporal sequence was captured at the molecular and clinical levels.

Life-course Mendelian randomisation provides genetic-instrumental support for the same temporal directionality. Urquijo et al. [12] showed that childhood adiposity raises paediatric asthma risk modestly (OR 1.20), but adulthood adiposity raises adult-onset asthma risk substantially (OR 1.37, *p* = 7 × 10^−12^). Childhood adiposity attenuates after adjustment for adult BMI, implying that adult adiposity is the proximal cause and that childhood adiposity acts through its predictive power for adult adiposity. Sun’s bidirectional analysis [11] independently excluded reverse causation. Genetic instruments for BMI are, by virtue of their prenatal allocation, temporally upstream of any asthma outcome. We recorded the year of obesity onset and the year of asthma diagnosis at recruitment in our own study. In the non-allergic-asthma-with-obesity group, the median duration of obesity was 9 years, substantially exceeding the median duration of asthma at 5 years, providing patient-level evidence consistent with obesity preceding asthma in this phenotype [7]. These particular findings are the authors’ own and are presented as such; the direction of the associations they describe is, however, independently supported by the Swiss SAPALDIA cohort [50], the Herrera-Luis epigenome-wide studies [20,21], the Hoang Agricultural Lung Health Study [67] and the Qatari cohort of Shailesh et al. [48], none of which involved the present authors. It should also be stated plainly that these are data from a single regional population—Mexican adolescents—so that their generalisability to other ancestries and settings remains to be established; the concordant findings in the Swiss, U.S. and Qatari cohorts are important precisely because they extend the signal beyond the authors’ own regional sample.

### 3.5. Biological Gradient (Dose–Response)

A monotonic dose–response relationship strengthens a causal interpretation. For obesity and asthma, dose–response relationships are observed both clinically and at the molecular level. Clinically, the Parasuaraman 2023 meta-analysis [16] provides the most authoritative recent dose–response evidence, showing a monotonic increase in risk across BMI categories, with separate gradients for waist circumference, abdominal fatness, and weight gain. At the molecular level, Shin et al. [51] reported a positive correlation between BMI and serum TNFα in adolescents (r = 0.39, *p* < 0.01). Schindler et al. [52] reported a positive correlation between BMI and Th17 cell frequency in peripheral blood (r = 0.42, *p* = 0.0005). Marijsse et al. [68] reported a positive correlation between BMI and IL17A messenger RNA expression in induced sputum (r = 0.22, *p* = 0.009). The Mendelian randomisation estimate reported by Mikkelsen et al. [15]—an RR of 1.05 per 1 kg/m^2^ increase in BMI—is itself a dose–response estimate based on genetic-instrumental variables and is concordant in direction and broadly similar in magnitude to the observational gradients.

### 3.6. Biological Plausibility

Plausibility is the viewpoint to which most contemporary research has been devoted, and it is also where the case for causation is strongest. The mechanistic chain is traceable from adipose tissue to the bronchial epithelium, spanning 12 distinct yet interacting layers—cellular, cytokine, transcriptional, epigenetic, effector, mechanical, adipokine, oxidative-metabolic, microbial, inflammasome, sex-hormonal, and structural—each supported by recent (2019–2026) human or translational evidence. We summarise the core immunological chain in detail and the ancillary layers more briefly.

The mechanistic chain begins in the adipose tissue itself. In sustained positive energy balance, existing adipocytes first enlarge (hypertrophy), and, as the limits of lipid storage within a given depot are approached, preadipocytes are recruited into adipogenic differentiation (hyperplasia) under the control of the transcription factors peroxisome proliferator-activated receptor γ (PPARγ) and CCAAT/enhancer-binding protein α [69,70]. Hypertrophic adipocytes outgrow their local capillary supply: intercapillary distances increase, regions of relative hypoxia develop, and stabilisation of hypoxia-inducible factor 1α drives the transcription of pro-inflammatory and pro-fibrotic genes, while adipocyte endoplasmic reticulum stress activates the JNK and IKKβ/NF-κB pathways [71]. Three consequences follow. Adipocytes secrete chemokines, principally CCL2 (monocyte chemoattractant protein-1); they release non-esterified fatty acids that act as TLR4 ligands on resident myeloid cells; and, where hypertrophy is extreme, individual adipocytes undergo necrosis, leaving residual lipid droplets that become encircled by macrophages in the crown-like structures characteristic of obese human adipose tissue [72,73]. Adipose tissue in obesity is therefore not simply larger tissue; it is tissue in which expansion has outstripped vascular and secretory capacity, and it is this mismatch, rather than fat mass as such, that generates the inflammatory signal.

Macrophage accumulation follows directly from these signals. In lean adipose tissue, resident macrophages are sparse and dispersed through the interstitium; in obesity, their number rises steeply, driven both by recruitment of circulating CCR2^+^ monocytes along the CCL2 gradient and by proliferation of macrophages already resident in the tissue [72,74]. Recruited cells adopt a classically activated (M1) programme under the combined influence of TLR4 ligation by saturated non-esterified fatty acids, local hypoxia, interferon-γ released by adipose-resident Th1 and CD8^+^ T cells, and leptin, which signals through the long isoform of its receptor to promote glycolytic reprogramming and NF-κB-dependent transcription. M1 macrophages secrete TNFα, IL-1β, IL-6, IL-15 and IL-23, and the abundance of these cytokines correlates with body mass index [53]. Periyalil et al. [54] showed that obese asthmatic adults have approximately twice the M1 macrophage density in visceral adipose tissue of obese non-asthmatic controls, which localises the difference to the asthma phenotype rather than to adiposity alone. Wang Y et al. [55] extended the picture to the airway: leptin-driven M1 polarisation produces elevated CXCL2. This soluble signal recruits neutrophils to the bronchial mucosa and thereby connects the adipose compartment to the effector site.

The corresponding change in the alternatively activated (M2) compartment is equally important and is frequently underemphasised. In lean adipose tissue, the resident population is predominantly M2, sustained by IL-4 and IL-13 derived from eosinophils and type 2 innate lymphoid cells and maintained transcriptionally through STAT6 and PPARγ/PPARδ signalling [75,76]. These cells restrain inflammation, support insulin sensitivity and clear apoptotic adipocytes. Obesity therefore produces not merely an accumulation of M1 cells but a shift in the M1:M2 ratio, since the adipose eosinophil and type 2 innate lymphoid cell populations that sustain the M2 programme contract as adiposity increases [76]. The consequences for the airway are twofold. First, in classical type 2 (allergic) asthma, M2 polarisation is itself a feature of the disease: IL-4 and IL-13 from Th2 cells and type 2 innate lymphoid cells induce airway macrophages to express arginase-1, CCL17 and CCL22, which amplify eosinophil and Th2 recruitment. In the non-allergic, obesity-associated phenotype, this axis is comparatively quiescent, and the airway macrophage compartment is skewed towards M1, which is consistent with the neutrophilic rather than eosinophilic inflammatory pattern that characterises the phenotype clinically [4,6,9]. Second, because efficient efferocytosis and the generation of specialised pro-resolving mediators are M2-dependent functions, relative loss of the M2 programme impairs the resolution phase of airway inflammation; impaired clearance of apoptotic cells has been demonstrated directly in obese asthma [77]. The M1:M2 imbalance thus contributes to the phenotype twice over by amplifying initiation and by retarding resolution. It should be added that the M1/M2 dichotomy is a useful abstraction rather than a description of discrete lineages; single-cell studies have resolved adipose and airway macrophages into a spectrum of states, including lipid-associated and metabolically activated populations that map cleanly onto neither pole [22,31].

The cytokines secreted by M1 macrophages (IL-1β, IL-6, IL-15, IL-23) are precisely those that drive naïve CD4^+^ T-cell differentiation into Th17 effectors, producing a positive feedback loop in which TNFα and IL-17A are reciprocally induced [56]. Pinkerton et al. [22] showed in 2022 that type 2 cytokine and inflammasome responses interact in the obese airway in ways that classical T2-dominated asthma models do not predict.

At the transcriptional level, RORC (retinoid-related orphan receptor C; RORγt in mouse) is the master transcription factor for Th17 differentiation [57]. Its expression is induced by both classical cytokine signalling—IL-6 acting through STAT3—and by metabolic ligands. The metabolic ligand pathway, characterised by Endo et al. [58], involves acetyl-CoA carboxylase 1 (ACC1), an enzyme of fatty acid biosynthesis whose expression is upregulated in obesity; ACC1-derived monounsaturated fatty acids and oxysterols act as ligand-dependent activators of RORC, providing a direct biochemical link between adiposity and Th17 polarisation. The dual routes to RORC activation—cytokine- and metabolite-mediated—explain why Th17 polarisation is particularly pronounced in obesity. Active RORC, with the histone acetyltransferase coactivator p300, binds the chromatin of Th17 lymphocytes and stimulates expression of *IL17A*, *IL17F* and *IL23R* [57].

The epigenetic layer is considered the most informative for explaining the durability of the phenotype. Mazzoni et al. [59] demonstrated that the methylation profile of the *RORC* and *IL17A* promoters is markedly lower in differentiated Th17 cells than in naïve CD4^+^ cells, establishing demethylation as a structural feature of the Th17 lineage. In 2020, it was hypothesised that obesity-driven hypomethylation of the *TNFA* promoter in M1 macrophages and of the *IL17A* and *RORC* promoters in Th17 lymphocytes constitutes the epigenetic mechanism by which obesity sustains the non-atopic asthma phenotype [38]. This hypothesis was directly tested in 2022 [8]: in 102 Mexican adolescents, methylation-specific quantitative PCR in peripheral blood leukocytes showed that *RORC* promoter methylation was significantly lower in the non-allergic-asthma-with-obesity group (median 43.9%, IQR 40.9–48.2) than in healthy controls (54.8%; *p* < 0.001) or in obesity without asthma (50.6%; *p* < 0.01); *IL17A* and *TNFA* methylation showed similar reductions. Importantly, *RORC* methylation was inversely correlated with both *RORC* (r_s_ = −0.39, *p* < 0.001) and *IL17A* (r_s_ = −0.37, *p* < 0.01) messenger RNA expression. These coupled methylation–expression correlations provide strong evidence that DNA methylation is the proximal regulator of the elevated transcript levels characterising the non-allergic-asthma-with-obesity phenotype. Replication is provided by the Swiss SAPALDIA EWAS [50], the Herrera-Luis EWAS in Hispanic/Latino youth [20], the Herrera-Luis EWAS in African-American and Hispanic youth [21], and the Hoang Agricultural Lung Health Study EWAS, in which 98.5% of differentially methylated CpGs in non-allergic adult asthma were hypomethylated relative to healthy controls [67]. The directionality caveat from Wahl et al.—that many BMI-associated methylation differences are consequences rather than causes of adiposity [78]—does not contradict the proposed pathway, as the sequence is obesity → systemic inflammation/methylation changes → asthma; the methylation marks are downstream of obesity but upstream of asthma. The longitudinal SAPALDIA design, in which increases in BMI precede changes in methylation, provides temporal anchoring [50].

**Figure 2:**
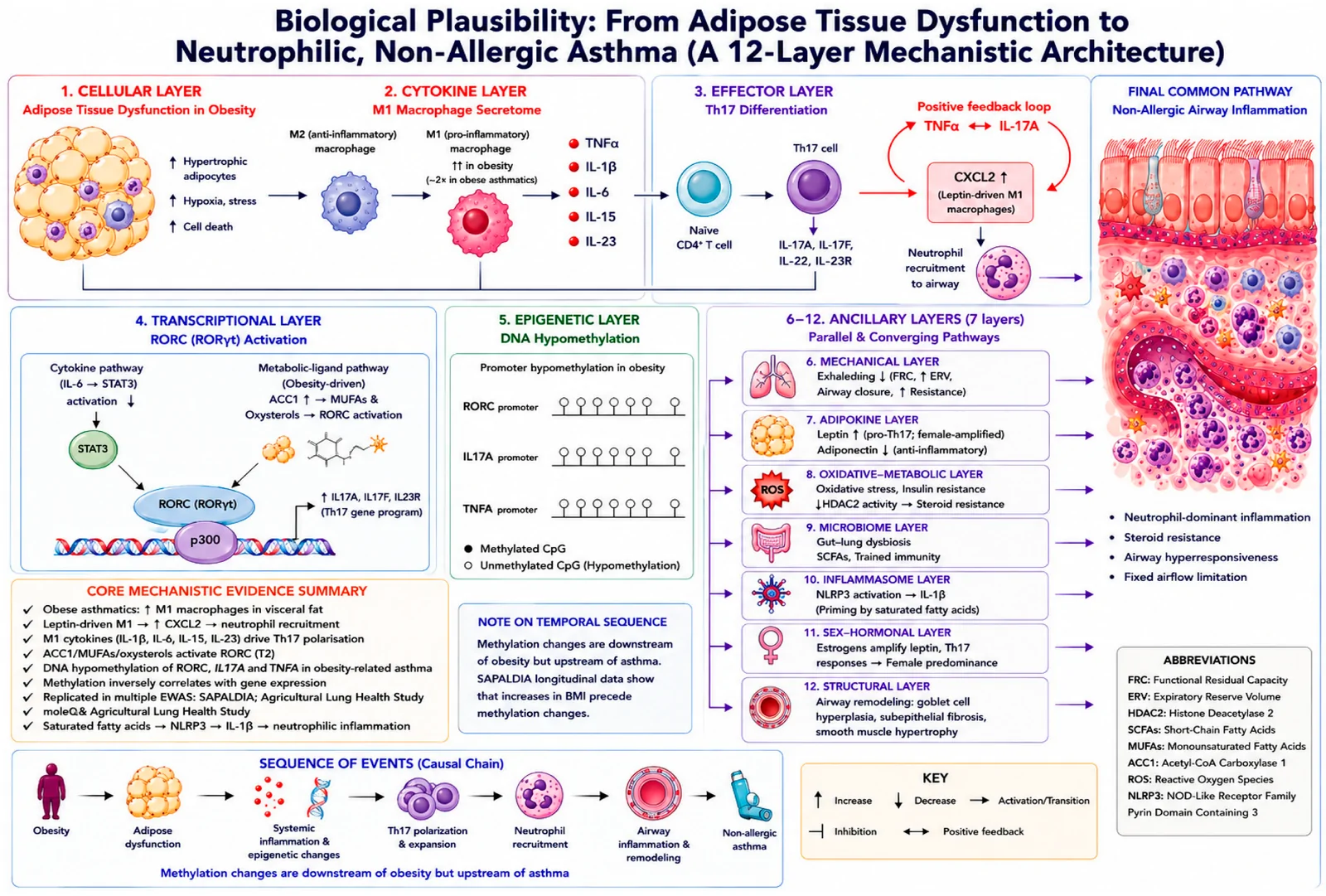
Biological plausibility of obesity-associated non-allergic asthma: a 12-layer mechanistic architecture. The figure summarises the proposed biological pathway linking adipose tissue dysfunction in obesity with neutrophilic, non-allergic asthma. The sequence begins with adipocyte hypertrophy, hypoxia, cellular stress and macrophage polarisation from an anti-inflammatory M2 phenotype toward a pro-inflammatory M1 phenotype. M1 macrophages release TNFα, IL-1β, IL-6, IL-15 and IL-23, which promote naïve CD4^+^ T-cell differentiation into Th17 cells and generate a reinforcing TNFα–IL-17A inflammatory loop. Leptin-driven M1 polarisation and CXCL2 production further promote neutrophil recruitment to the airway. At the transcriptional level, RORC/RORγt activation occurs through both cytokine-mediated IL-6–STAT3 signalling and obesity-driven metabolic ligand pathways involving ACC1, monounsaturated fatty acids and oxysterols. Epigenetically, hypomethylation of the RORC, IL17A and TNFA promoters supports sustained inflammatory gene expression, positioning methylation changes downstream of obesity but upstream of asthma expression. The first five layers represent the core immunological pathway—cellular, cytokine, effector, transcriptional and epigenetic—whereas the seven ancillary layers—mechanical, adipokine, oxidative-metabolic, microbial, inflammasome, sex-hormonal and structural—complete the 12-layer architecture and converge on the final common pathway of non-allergic airway inflammation. Abbreviations: ACC1, acetyl-CoA carboxylase 1; BMI, body mass index; CXCL2, C-X-C motif chemokine ligand 2; ERV, expiratory reserve volume; FRC, functional residual capacity; HDAC2, histone deacetylase 2; IL, interleukin; M1, macrophage type 1 (pro-inflammatory); M2, macrophage type 2 (anti-inflammatory); MUFAs, monounsaturated fatty acids; NLRP3, NOD-like receptor family pyrin domain containing 3; RORC, retinoid-related orphan receptor C; RORγt, retinoid-related orphan receptor gamma t; SCFAs, short-chain fatty acids; STAT3, signal transducer and activator of transcription 3; Th17, T-helper 17 cell; TNFα, tumour necrosis factor alpha.

The remaining ancillary layers reinforce the core chain. Mechanical fat loading reduces functional residual capacity and expiratory reserve volume, with peripheral airway closure and oscillometric heterogeneity in resistance and compliance documented in obese asthmatic adults [79,80]. Adipokine biology contributes through leptin (pro-Th17, sex-stratified, female-amplified) and adiponectin (anti-inflammatory, reduced in obesity). Oxidative stress and insulin resistance reduce histone deacetylase 2 activity and contribute to corticosteroid resistance. Gut–lung microbial axis dysbiosis modulates short-chain fatty acid signalling and trained innate immunity. The NLRP3 inflammasome and ILC3-derived IL-17A constitute a parallel innate-immunological route to neutrophilic effector inflammation; the 2025 McCright et al. paper [23] in Science Translational Medicine showed in mice and humans that dietary saturated fatty acids drive lung-macrophage NLRP3 priming and IL-1β-mediated neutrophil-predominant inflammation, with the same monocyte signature identified in obese humans with asthma. Sex-hormonal modulation explains the female predominance via oestrogen-driven adipose accumulation, which increases leptin levels and amplifies Th17 polarisation. Airway remodelling—epithelial and sub-epithelial alterations, smooth-muscle hyperplasia, increased airway vascularity—represents the durable structural correlate of the chronic inflammatory state [30]. The plausibility argument is therefore not a single argument but a stack of mutually reinforcing arguments distributed across twelve layers—cellular, cytokine, transcriptional, epigenetic, effector, mechanical, adipokine, oxidative-metabolic, microbial, inflammasome, sex-hormonal and structural—each supported by recent (2019–2026) human or translational evidence. These twelve layers and their interrelations are depicted in Figure 2.

### 3.7. Coherence

Coherence asks whether the proposed causal interpretation is consistent with the broader body of knowledge about the disease’s natural history and biology. For the obesity–non-T2 asthma association, the answer is largely affirmative.

The late-onset obese asthma phenotype is characterised by greater asthma severity, neutrophilic rather than eosinophilic airway inflammation, female predominance, and reduced responsiveness to inhaled corticosteroids [4,6,9]. Each of these clinical features correlates with the proposed Th17/neutrophilic mechanism. Steroid resistance is itself molecularly coherent: glucocorticoids do not suppress IL-17A production in vitro [10]. Liu et al. [6], in 2024, in the European Respiratory Journal, reported sputum neutrophilia in obese asthma at 52% versus 36% in lean asthma, with cluster analyses confirming that neutrophilic non-T2 phenotypes are more severe. The 2024 American Journal of Respiratory and Critical Care Medicine state-of-the-art review by Althoff et al. [29] consolidates the mechanism–treatment link specifically for obesity-related asthma. A point of partial incoherence—anti-IL-17 trial nulls in unselected severe asthma populations [60]—most plausibly reflects the absence of biomarker-based selection for obese, neutrophilic, IL-17A-high subgroups rather than a flaw in the underlying causal model.

### 3.8. Experimental Evidence

Hill identified experimental evidence as the most decisive of his nine viewpoints. For obesity and asthma, true randomised allocation to obesity is impossible; experimental evidence comes instead from four converging streams: weight loss interventions (bariatric surgery and lifestyle), GLP-1 receptor agonist pharmaco-epidemiology, animal models, and pharmacological blockade of the implicated pathway.

For obesity and asthma, experimental evidence comes from four converging streams. First, weight loss interventions provide the closest analogue to a clinical experiment. The 2023 systematic review and meta-analysis of 15 studies by Xie et al. [61] showed consistent pre- and post-bariatric surgery reductions in asthma medication use; Smith et al. [62] reported a durable 5-year benefit in 2024. Sharma et al. [63], in 2025, in Chest, reported a randomised controlled trial of a counterweight-based programme in difficult-to-treat asthma with obesity, demonstrating clinically meaningful improvements in asthma control and lung function with formula-based total diet replacement.

Second, glucagon-like peptide-1 receptor agonists provide the most informative quasi-experimental modifier. Foer et al. [24] in 2021 used an electronic health records cohort to show that GLP-1RA initiators (reference) had substantially fewer asthma exacerbations than initiators of comparator antihyperglycaemic drugs (incidence rate ratios of 1.83–2.98). Wang T et al. [25] in 2024 confirmed heterogeneous treatment effects, with the strongest benefit in obese patients with prior exacerbations. Huang YC et al. [26], in JAMA Network Open in 2025, reported that among TriNetX-matched adolescents with concurrent asthma and overweight/obesity, GLP-1RA users had 49% fewer asthma exacerbations, 58% fewer emergency department visits, and 34% lower systemic corticosteroid use. The Vanderbilt-led GATA-3 randomised controlled trial (NCT05254314) is in progress, with results expected in 2026. These data are, however, predominantly pharmaco-epidemiological and therefore open to residual confounding by indication and to healthy-adherer effects; the causal reading of the GLP-1 receptor agonist signal should be regarded as provisional until the dedicated randomised trial reports.

Third, pharmacological modulation of the proposed effector pathway provides direct experimental evidence. Tezepelumab—the only biologic approved for non-T2 asthma—reduced annualised exacerbation rates by 48% in patients with baseline blood eosinophils <150 cells/μL and 40% in those with FeNO < 25 ppb in pooled NAVIGATOR + PATHWAY analyses [81,82]. Anti-IL-17 trials (brodalumab, CJM112, risankizumab) [60] enrolled unselected severe asthma populations without biomarker-based selection for obese, neutrophilic, IL-17A-high subgroups; biomarker-stratified trials in the obese asthma endotype are warranted. Fourth, animal models reproduce the phenotype: Mathews et al. [39] showed in C57BL/6J mice on a high-fat diet that IL-17A elevation precedes the development of airway hyperresponsiveness, providing temporal precedence at the mechanistic level. The interpretation that these null results reflect the absence of biomarker-based enrichment, rather than a genuine absence of effect, is biologically plausible but remains a hypothesis, since no adequately powered biomarker-stratified trial in the obese, neutrophilic, IL-17A-high endotype has yet been completed. Biomarker selection is, moreover, unlikely to be the only explanation. Even in a correctly enriched population, redundancy within the type-17 and neutrophilic programme could blunt the effect of blocking IL-17A alone: innate sources of the same effector cytokines—ILC3-derived IL-17 and IL-22—and parallel neutrophil-recruiting axes such as IL-1β–IL-23 and IL-8/CXCR2 can sustain airway neutrophilia through routes that partly bypass adaptive IL-17A, so that single-cytokine blockade may be insufficient even where the endotype is correctly identified. This broader pathophysiological possibility—pathway redundancy and alternative innate mechanisms—should be weighed alongside the biomarker selection hypothesis rather than treated as subordinate to it.

### 3.9. Analogy

The TNFα/Th17 axis driving obesity-related asthma also drives obesity-related psoriasis, inflammatory bowel disease, rheumatoid arthritis, non-alcoholic fatty liver disease and type 2 diabetes mellitus [31]. A new analogy stream has emerged in the GLP-1 receptor agonist literature: the same agents that reduce asthma exacerbations in obesity reduce major adverse cardiovascular events, ameliorate non-alcoholic steatohepatitis, and have signalled benefit in chronic obstructive pulmonary disease pharmaco-epidemiology. The shared metabolic–inflammatory mechanism across diseases makes the analogy informative.

## 4. The 1964 Surgeon General Antecedent, GRADE and Contemporary Causal-Inference Methods

Hill’s framework descends directly from the 1964 U.S. Surgeon General’s Report on Smoking and Health [34], which articulated five criteria—consistency, strength, specificity, temporality and coherence—for inferring causation from epidemiological evidence. Hill expanded these to nine in his 1965 address by adding biological gradient, plausibility, experiment and analogy, reflecting his concern that observational epidemiology be brought into closer dialogue with biological mechanism and quantitative regularities. Subsequent Surgeon General’s Reports introduced a four-tier classification of the strength of causal inference (sufficient evidence, suggestive but not sufficient, inadequate, suggestive of no causal relationship). Applied to the obesity–non-T2 asthma association, our reading of the cumulative 2020–2026 evidence corresponds most closely to the second tier (“suggestive but not yet sufficient”) for the strict causal claim that obesity causes the late-onset, non-allergic asthma phenotype and to the first tier (“sufficient evidence”) for the weaker claim that obesity is causally associated with increased risk of incident asthma in general.

The GRADE framework [35] complements rather than replaces Hill’s viewpoints. Hill asks: should this association be interpreted as causal? GRADE asks: how confident should I be in the magnitude and direction of this effect estimate when using it for clinical decisions? In the GRADE schema, observational evidence starts at “low” certainty and can be upgraded for large effect sizes, dose–response, or the directionality of plausible residual confounding. For the obesity–non-T2 asthma association, several upgrade criteria are met: some paediatric estimates exceed the conventional large-effect threshold; a graded dose–response is documented at population, molecular and genetic-instrumental levels; and the directionality of plausible residual confounding (socioeconomic position, dietary pattern, physical activity) is conservative, biasing the association toward rather than away from the null. A reasonable summary GRADE assessment of the body of evidence for obesity → incident asthma is therefore of moderate certainty.

Three further methodological developments are particularly relevant. The counterfactual or potential outcomes framework [36,37] forces the investigator to specify exactly which hypothetical intervention is being modelled—in this case, the average causal effect of a population-wide intervention on asthma incidence that maintains BMI below 25 kg/m^2^ throughout adulthood. Directed acyclic graphs (DAGs) [36] visually encode causal assumptions and clarify confounders, mediators, and colliders—essential for the obesity–asthma question because adjustment for body composition mediators (visceral fat, leptin, insulin resistance) when estimating the total effect produces biased estimates. Mendelian randomisation [33], in which genetic variants associated with BMI are used as instrumental variables, is the most consequential development for the obesity–asthma question: six MR studies of particular relevance [11,12,13,14,15] jointly transform the inferential picture, with phenotype-specific amplification of the signal in non-atopic, adult-onset and female-predominant subgroups and consistent exclusion of reverse causation. A methodological gap remains: no MR study has yet stratified asthma by the non-T2/late-onset/neutrophilic subtype with BMI as the exposure. Phenotype-specific MR is now the most decisive missing piece of the causal argument.

**Figure 3:**
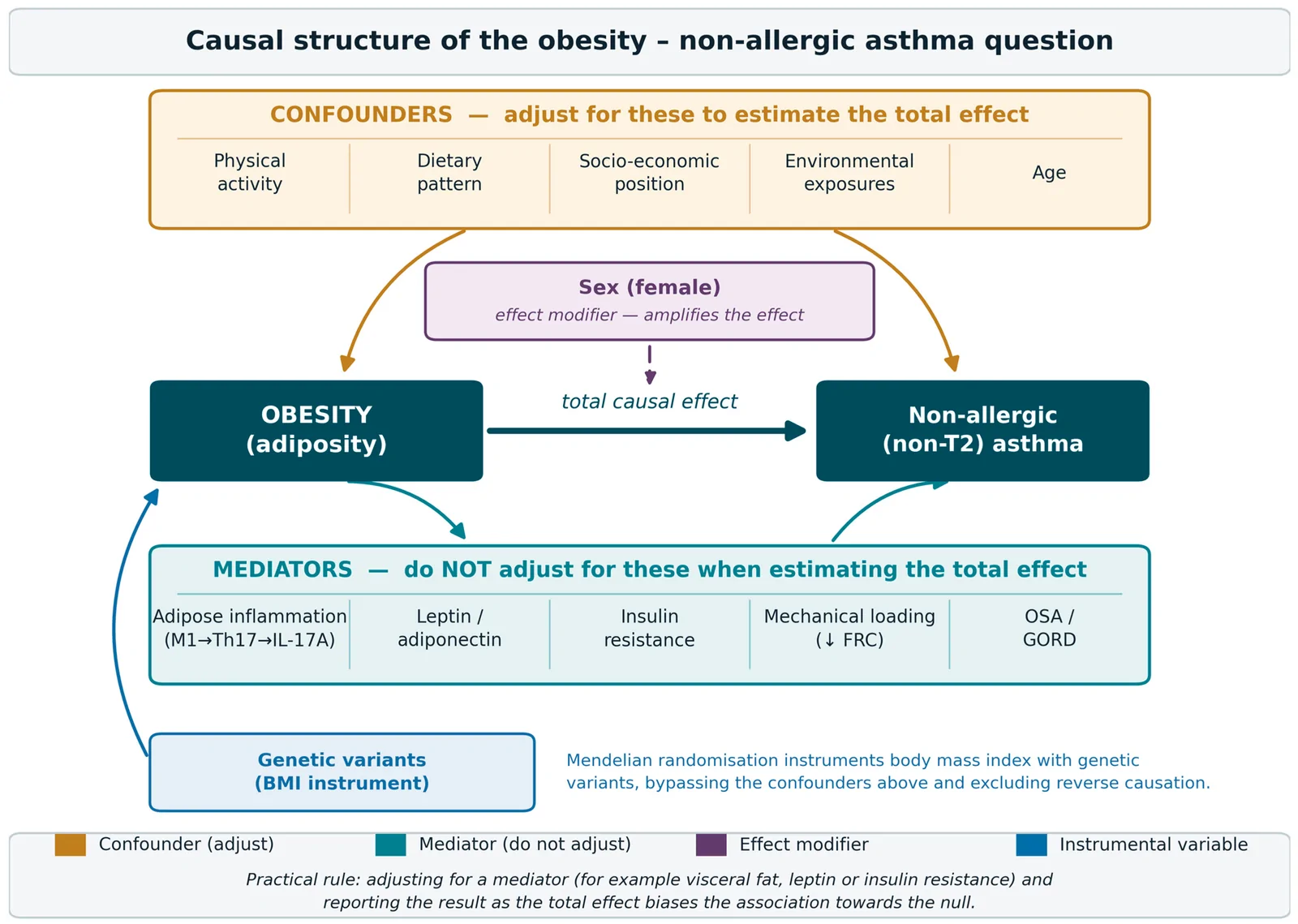
A template directed acyclic graph (DAG) for the obesity → non-allergic asthma question. Confounders—the common causes of both obesity and asthma—should be adjusted for when the total effect is estimated; mediators, which lie on the causal path, should not, because conditioning on them removes part of the effect under study and biases the estimate towards the null. Sex is shown as an effect modifier and genetic variants as the instrumental variable exploited by Mendelian randomisation. Abbreviations: FRC, functional residual capacity; GORD, gastro-oesophageal reflux disease; IL-17A, interleukin-17A; M1, macrophage type 1 (pro-inflammatory); OSA, obstructive sleep apnoea; Th17, T-helper 17.

The distinction between confounders, mediators and effect modifiers is central to this question and is made explicit in Figure 3. Obesity shares common causes with asthma—physical inactivity, dietary pattern, socio-economic position, environmental exposures such as indoor allergens and air pollution, and age—and these act as confounders that must be adjusted for, or otherwise accounted for, when the total effect of obesity is estimated; residual confounding by this set is the principal threat to the observational estimates, and it is precisely this threat that the E-values quantify and that Mendelian randomisation circumvents by instrumenting body mass index with genetic variants. A second set of variables—visceral adipose inflammation and its downstream M1–Th17–IL-17A axis, leptin and adiponectin, insulin resistance, mechanical loading of the chest wall, and the obesity-associated comorbidities obstructive sleep apnoea and gastro-oesophageal reflux disease—lies on the causal path from obesity to asthma. These are mediators, not confounders: conditioning on them when the total effect is the target blocks part of the very effect under study and biases the estimate towards the null, which is one reason why analyses that adjust heavily for metabolic covariates tend to report attenuated associations. Sex is best treated as an effect modifier rather than a confounder, the effect being consistently stronger in females [15,19,42]; medications and healthcare utilisation may act as confounders or as colliders depending on the analysis and require case-by-case consideration. The practical consequence for readers appraising this literature is that an effect estimate is interpretable only once its adjustment set is known: an estimate adjusted for mediators answers a different question (the direct effect) from one adjusted only for confounders (the total effect), and the two should not be compared as though they were the same quantity. Obstructive sleep apnoea deserves particular mention in this respect: its relationship with asthma is bidirectional—obesity promotes both conditions, sleep apnoea aggravates asthma control through intermittent hypoxia and systemic inflammation, and poorly controlled asthma in turn worsens sleep-disordered breathing [83]—so that it is best modelled as a mediator that also exerts feedback on the outcome rather than as a simple upstream confounder.

## 5. Synthesis and Implications

Table 3 draws the nine viewpoints together, stating for each its status, the principal findings with their supporting data, and the corresponding references.

**Table 3:** Summary of the nine Bradford Hill viewpoints applied to obesity → non-allergic asthma: status of the evidence, principal findings with supporting data, and key references (numbering as in the reference list).

Viewpoint	Status	Principal Findings and Supporting Data	References
1. Strength	Met (moderate to large)	Adult cohorts and meta-analyses: OR/RR 1.32–2.70 Paediatric estimates: OR/RR 1.40–6.80, largest in girls E-values 4.8 and 13.1 exceed the strength of any plausible single confounder Mendelian randomisation: summary RR 1.05 per 1 kg/m^2^	[15,16,17,18,19,32,40,41,42,43,44,45]
2. Consistency	Met	Replicated in the USA, Northern Europe, Asia and Latin America Inflammatory pattern reproduced in Qatar (364 children) U-BIOPRED cluster T4 replicated in the Chinese C-BIOPRED cohort Female predominance refines rather than undermines consistency Early-childhood null findings fit the life-course pattern	[16,17,18,28,42,43,46,47,48,64,65,66]
3. Specificity	Met at the phenotype level	MR: non-atopic asthma OR 1.42–1.72 vs. atopic 1.18–1.26 per 1 SD BMI Cluster analysis isolates a late-onset, non-allergic phenotype Th1- rather than Th2-dominant immune response (meta-analysis) RORC mRNA discriminates the phenotype: AUC 0.95 (0.86–0.99)	[4,7,9,11,13,49]
4. Temporality	Met (sine qua non)	Obesity precedes asthma in three independent prospective cohorts SAPALDIA: rising BMI precedes methylation change over 10 years Life-course MR: adult adiposity → adult-onset asthma, OR 1.37 (*p* = 7 × 10^−12^) Bidirectional MR excludes reverse causation Patient level: median obesity duration 9 years vs. asthma 5 years	[7,11,12,17,40,42,50]
5. Biological gradient	Met	RR 1.32 per 5 kg/m^2^ BMI; 1.26 per 10 cm waist; 1.33 per 10 kg weight gain BMI vs. serum TNFα: r = 0.39 (*p* < 0.01) BMI vs. peripheral Th17 frequency: r = 0.42 (*p* = 0.0005) BMI vs. sputum IL17A mRNA: r = 0.22 (*p* = 0.009) Genetic dose–response: RR 1.05 per 1 kg/m^2^	[15,16,51,52,68]
6. Plausibility	Strongly met (12 layers)	Adipose dysfunction → M1 polarisation → CXCL2 → airway neutrophilia M1↔Th17 positive feedback loop (TNFα ↔ IL-17A) Dual RORC activation: IL-6/STAT3 and ACC1-derived metabolic ligands RORC promoter methylation 43.9% vs. 54.8% in controls (*p* < 0.001) Methylation inversely correlated with RORC (r_s_ = −0.39) and IL17A (r_s_ = −0.37) mRNA Seven ancillary layers: mechanical, adipokine, oxidative-metabolic, microbial, inflammasome, sex-hormonal, structural	[8,20,21,22,23,30,31,38,53,54,55,56,57,58,59,67,69,70,71,72,73,74,75,76,77,78,79,80]
7. Coherence	Met (with caveats)	Phenotype is neutrophilic, steroid-resistant, female-predominant and severe Sputum neutrophilia 52% in obese vs. 36% in lean asthma Corticosteroids do not suppress IL-17A production in vitro Caveat: anti-IL-17 trials null in unselected severe-asthma populations	[4,6,9,10,29,60]
8. Experiment	Met (four streams)	Bariatric surgery: consistent reduction in asthma medication across 15 studies, durable at 5 years Randomised weight loss programme improved control and lung function (2025) GLP-1RA in adolescents: 49% fewer exacerbations, 58% fewer ED visits, 34% less systemic corticosteroid Tezepelumab reduces exacerbations by 40–48% in T2-low subgroups Diet-induced obese mice: IL-17A rise precedes airway hyperresponsiveness	[24,25,26,39,60,61,62,63,81,82]
9. Analogy	Met	Same TNFα/Th17 axis in psoriasis, IBD, rheumatoid arthritis, MASLD and type 2 diabetes GLP-1 receptor agonist benefit shared across obesity-related inflammatory disease	[24,25,26,31]

Abbreviations: AUC, area under the receiver operating characteristic curve; BMI, body mass index; GLP-1RA, glucagon-like peptide-1 receptor agonist; IBD, inflammatory bowel disease; MASLD, metabolic dysfunction-associated steatotic liver disease; MR, Mendelian randomisation; OR, odds ratio; RORC, retinoid-related orphan receptor C; RR, relative risk; r_s_, Spearman’s rank correlation coefficient; SD, standard deviation; T2, type 2; ↔, indicates bidirectional crosstalk.

Taken together, the evidence reviewed here strongly supports a causal contribution of obesity to the development of a distinctive, non-allergic, late-onset asthma phenotype in susceptible individuals, without amounting to definitive proof. A distinction must be maintained throughout: the largest and most consistent quantitative estimates (Parasuaraman [16]; the Mendelian randomisation summary [15]) concern obesity and incident asthma in general, whereas the phenotype-specific claim rests on cluster-analytical, immunological and single-cohort Mendelian randomisation evidence [4,7,11]. No Mendelian randomisation study has yet used the non-T2, late-onset, neutrophilic subtype itself as the outcome, so the phenotype-level causal claim remains one inferential step beyond the directly instrumented evidence. This argument is based on prospective cohort and life-course Mendelian randomisation evidence establishing temporality (Camargo [40], Castro-Rodríguez [42], Vartiainen [17], SAPALDIA [50], Sun [11], Urquijo [12]), updated meta-analytic evidence establishing strength and consistency (Parasuaraman [16], Bloodworth [18], Chen [19]), and molecular and epigenetic evidence establishing biological plausibility across 12 layers. Central mechanisms include the M1–Th17 feedback loop, the RORC transcriptional network, the ACC1-mediated metabolic ligand pathway, and the epigenetic remodelling of the RORC, IL17A, and TNFA promoters. Additional support comes from murine and in vitro mechanistic evidence, GLP-1 receptor agonist pharmaco-epidemiology, bariatric/lifestyle weight loss data, and clinical analogy with other obesity-related Th17 diseases. All nine Hill viewpoints are satisfied to varying degrees: strength, consistency, biological gradient, plausibility, coherence, experiment, and analogy substantively; specificity at the phenotype level; and temporality as a sine qua non.

Three primary implications emerge. First, from a public health perspective, preventing obesity in childhood and adolescence should be prioritised as a primary prevention strategy for the late-onset, non-T2 asthma phenotype, particularly in middle-income countries experiencing sharp increases in childhood and adolescent obesity prevalence. The Mexican prevalence figures from ENSANUT 2022 [27] (overweight: 38.3%; obesity: 36.9%; abdominal obesity: 81.0% in adults; rising sedentary behaviour and BMI in school-age and adolescent populations) provide relevant context. The life-course MR finding that adult adiposity drives adult-onset asthma [12] underscores the importance of paediatric prevention, as childhood and adolescent obesity is the strongest predictor of sustained adult adiposity. Second, in therapeutic terms, this phenotype necessitates endotype-targeted strategies, including GLP-1 receptor agonists (with the GATA-3 RCT pending), tezepelumab for non-T2 asthma, and biomarker-stratified anti-IL-17 strategies. The 2021 finding that RORC mRNA expression discriminates non-allergic asthma among obese adolescents, with an AUC of 0.95 [7], indicates that RORC transcriptional readouts, possibly combined with promoter methylation analysis, may serve as the patient-selection biomarker that anti-IL-17 trials in obese asthma have lacked. Third, research priorities include longitudinal cohorts sampling BMI, immunological readouts, and methylation profiles at multiple time points; phenotype-stratified Mendelian randomisation studies; and single-cell methylation profiling of sorted peripheral immune cells.

## 6. Limitations and Methodological Gaps

Several limitations and methodological gaps must be acknowledged. First, the directionality of the BMI–methylation association is partly downstream rather than upstream [78]; the proposed pathway (obesity → systemic inflammation/methylation → asthma) aligns with this finding, and the SAPALDIA longitudinal design [50] provides temporal anchoring. However, the predominantly cross-sectional nature of the supporting epigenetic literature, including the authors’ studies [7,8,38], does not allow for directionality to be resolved at the individual level. Second, the cell of origin for the methylation signal in peripheral blood leukocytes remains undetermined; single-cell methylation profiling is the logical next step. Third, evidence for GLP-1 receptor agonists is primarily derived from pharmaco-epidemiological data, with the GATA-3 RCT as the first dedicated trial. Fourth, the anti-IL-17 trial nulls [60] present a significant challenge to the experimental viewpoint; biomarker-stratified trials in the obese asthma endotype are warranted but have not yet been conducted. Fifth, the most decisive Mendelian randomisation studies stratify by atopic versus non-atopic asthma but not by the molecular phenotypes addressed in this manuscript (non-T2, late-onset, neutrophilic, steroid-resistant). Phenotype-specific Mendelian randomisation analyses are now the most important missing element in the causal argument. Because Mendelian randomisation is treated here as a principal pillar of causal inference, this gap sets an explicit ceiling on the certainty that can be claimed. The available genetic-instrumental evidence licenses a confident causal statement about obesity and incident asthma in general and about the atopic–non-atopic contrast but only an indirect, mechanism-bridged inference about the specific non-T2, neutrophilic phenotype that is the subject of this review. Until a genetic instrument for adiposity is tested against a molecularly defined neutrophilic or non-T2 outcome, the phenotype-level conclusion cannot rise above the “suggestive but not sufficient” tier, however coherent the supporting observational and mechanistic evidence; it is the absence of this single experiment of nature, rather than any positive counter-evidence, that holds the narrow claim below the “sufficient” threshold.

## 7. Conclusions and Future Directions

Sixty years after Hill’s Presidential Address, his nine viewpoints continue to serve as the most effective organising framework for causal inference in observational respiratory epidemiology, provided they are interpreted as structured viewpoints rather than as a checklist. Systematic application of this framework to the 2020–2026 evidence base on obesity and the non-allergic, late-onset, neutrophilic asthma phenotype yields a coherent causal interpretation. The evidentiary landscape has been transformed in the past five years by bidirectional and life-course Mendelian randomisation analyses, GLP-1 receptor agonist pharmaco-epidemiology, translational epigenetic and single-cell evidence, and integrative clinical syntheses. On balance, it is appropriate to regard obesity as a probable cause of, or a strong causal contributor to, the late-onset, non-allergic, neutrophilic asthma phenotype—an interpretation that the cumulative evidence supports but does not yet definitively establish, consistent with the “suggestive but not sufficient” evidentiary tier assigned above, and with the phenotype-specific Mendelian-randomisation evidence still outstanding. Primary prevention of childhood and adolescent obesity, endotype-targeted therapeutics, including GLP-1 receptor agonists and biomarker-stratified anti-IL-17 strategies, and longitudinal triangulation of the causal model through phenotype-specific Mendelian randomisation studies and single-cell methylation profiling are recommended as next steps for the field.

## Figures and Tables

**Table 1:** Operational definition of the asthma phenotype under discussion, distinguishing the clinical phenotype, the inflammatory endotype and the biomarker profile, and contrasting it with the allergic (type 2) phenotype.

Level of Description	Non-Allergic, Obesity-Associated Phenotype (This Review)	Allergic/Type 2 Phenotype (Contrast)
Terminology and its meaning	Late-onset (timing); non-T2/non-Th2 (endotype); neutrophilic (airway cell); steroid-resistant (treatment response); obesity-related/-associated (aetiology). These are distinct descriptors, not synonyms.	Early-onset; T2/Th2; eosinophilic; corticosteroid-responsive; allergic/atopic.
Clinical phenotype	Onset typically after age 12 or in adulthood; female-predominant; higher body mass index; greater severity and more frequent exacerbations; obesity generally precedes asthma.	Onset usually in childhood; associated with allergic sensitisation; obesity, when present, is a later comorbidity of pre-existing asthma.
Inflammatory endotype	Non-type 2: Th17- and Th1-skewed adaptive response with M1 macrophages and ILC3; low type 2 signalling.	Type 2: Th2 and ILC2 response with IgE class-switching; high type 2 signalling.
Airway inflammation	Neutrophilic (sputum neutrophilia, ~52% vs. ~36% in lean asthma [6]); low eosinophils and low FeNO.	Eosinophilic; elevated FeNO.
Biomarker profile	↑ IL-17A, TNFα, IL-6, C-reactive protein, leptin; ↑ Th17 frequency; ↑ RORC mRNA; ↓ RORC-promoter methylation; low blood eosinophils and FeNO [4,7,8,9].	↑ total and specific IgE, blood and sputum eosinophils, FeNO, periostin; type 2 cytokines IL-4, IL-5, IL-13.
Treatment response and candidate targets	Relatively corticosteroid-resistant [10]; candidate strategies include weight loss, GLP-1 receptor agonists, tezepelumab, and biomarker-stratified anti-IL-17.	Corticosteroid-responsive; anti-IgE, anti-IL-5/5R and anti-IL-4Rα biologics.

Abbreviations: FeNO, fractional exhaled nitric oxide; ILC, innate lymphoid cell; IgE, immunoglobulin E; RORC, retinoid-related orphan receptor C; Th, T-helper. ↑ indicates an increase, and ↓ indicates a decrease.

## Data Availability

No new data were created or analyzed in this study. Data sharing is not applicable to this article.

## Supplementary Materials

The following supporting information can be downloaded at: https://www.mdpi.com/article/10.3390/epidemiologia7040107/s1, Search strategy: The complete search strings for the three databases are provided in the accompanying Supplementary Material. The searches were run in PubMed/MEDLINE, Scopus and the Web of Science Core Collection for the period 1 January 1995 to 1 June 2026, restricted to English and Spanish. The strategy was adapted from the authors’ registered paediatric systematic review protocol (PROSPERO CRD42023472442) by removing the paediatric and human-only restrictions and by adding a causal inference/study design block, so that adult and paediatric evidence and animal and translational studies—required for the experiment and analogy viewpoints—would be retrieved.
