# Supplementary material for: Causal Inference in Non-Allergic Asthma Associated with Obesity: Application of the Bradford Hill Viewpoints to a Complex Epidemiological Association
Source: Epidemiologia (Basel). 2026 Aug 6;7(4):107. doi: 10.3390/epidemiologia7040107 (PMC13512083; doi:10.3390/epidemiologia7040107)
Supplement: Supplementary file 1 [file epidemiologia-07-00107-s001.zip › epidemiologia-4380948-supplementary.pdf]

Supplementary Material — Search strategy

The searches below were run in PubMed/MEDLINE, Scopus and the Web of Science Core Collection for the period 1 January 1995 to 1 June 2026, restricted to the English and Spanish languages. The synthesis emphasises the evidence published between 2020 and 2026, while the full window captures the landmark earlier studies on which several Bradford Hill viewpoints depend. The strategy was adapted from the authors' registered paediatric systematic-review protocol (PROSPERO CRD42023472442) with two deliberate broadenings: (i) the paediatric restriction was removed, so that adult and paediatric evidence are both eligible; and (ii) the human-only restriction was removed, so that animal and in-vitro/translational studies are eligible — these are required for the experiment and analogy viewpoints and would have been excluded by a human-only filter. A causal-inference / study-design block was also added (Mendelian randomisation, cohort, dose-response, bariatric surgery, weight loss, GLP-1 receptor agonist, epigenome-wide).

PubMed / MEDLINE

```
#1  Exposure: "body fat distribution"[MeSH] OR "adipose tissue"[MeSH] OR "body mass index"[MeSH] OR overweight[MeSH] OR obesity[MeSH] OR adiposity[tiab] OR "waist circumference"[tiab] OR "visceral fat"[tiab]

#2  Outcome: asthma[MeSH] OR wheezing[tiab] OR "non-allergic asthma"[tiab] OR "non-atopic asthma"[tiab] OR "non-T2 asthma"[tiab] OR "non-Th2 asthma"[tiab] OR "neutrophilic asthma"[tiab] OR "late-onset asthma"[tiab] OR "obesity-related asthma"[tiab] OR "obesity-associated asthma"[tiab] OR "steroid-resistant asthma"[tiab]

#3  Mechanism: adipokines[MeSH] OR cytokines[MeSH] OR "C-reactive protein"[MeSH] OR neutrophils[MeSH] OR eosinophils[MeSH] OR "T-Lymphocytes, Helper-Inducer"[MeSH] OR Th17[tiab] OR "interleukin-17"[tiab] OR RORC[tiab] OR inflammation[MeSH] OR "DNA methylation"[MeSH] OR NLRP3[tiab]

#4  Causal inference / design: "Mendelian randomization"[tiab] OR "causal inference"[tiab] OR "Bradford Hill"[tiab] OR cohort[tiab] OR "dose-response"[tiab] OR "bariatric surgery"[MeSH] OR "weight loss"[MeSH] OR "glucagon-like peptide 1"[tiab] OR "GLP-1 receptor agonist"[tiab] OR "epigenome-wide"[tiab]

#5  (#1 AND #2) AND (#3 OR #4).  Filters: Publication date 1995/01/01:2026/06/01; Languages English, Spanish. No "Humans" filter and no age restriction were applied.
```

Web of Science Core Collection

```
#1  ALL=(body fat distribution OR adipose tissue OR body mass index OR overweight OR obesity OR adiposity OR waist circumference OR visceral fat)

#2  ALL=(asthma OR wheezing OR non-allergic asthma OR non-atopic asthma OR non-T2 asthma OR non-Th2 asthma OR neutrophilic asthma OR late-onset asthma OR obesity-related asthma OR obesity-associated asthma OR steroid-resistant asthma)

#3  ALL=(adipokines OR cytokines OR C-reactive protein OR neutrophil OR eosinophils OR T-helper OR Th17 OR interleukin-17 OR RORC OR inflammation OR DNA methylation OR NLRP3)

#4  ALL=(Mendelian randomization OR causal inference OR Bradford Hill OR cohort OR dose-response OR bariatric surgery OR weight loss OR glucagon-like peptide 1 OR GLP-1 receptor agonist OR epigenome-wide)

#5  (#1 AND #2) AND (#3 OR #4).  Timespan 1995–2026; document types Article and Review; languages English and Spanish. The child*/adult* and animal exclusions of the original protocol were removed.
```

Scopus

```
TITLE-ABS-KEY ( ( obesity OR overweight OR adiposity OR "body mass index" ) AND ( asthma OR "obesity-related asthma" OR "obesity-associated asthma" OR "non-allergic asthma" OR
```

```
"non-T2 asthma" OR "neutrophilic asthma" OR "late-onset asthma" ) AND ( "Mendelian
randomization" OR "causal inference" OR cohort OR "dose-response" OR "bariatric surgery"
OR "weight loss" OR "GLP-1 receptor agonist" OR cytokine OR interleukin OR neutrophil OR
"DNA methylation" ) ) AND PUBYEAR > 1994 AND PUBYEAR < 2027 AND ( LIMIT-TO ( DOCTYPE ,
"ar" ) OR LIMIT-TO ( DOCTYPE , "re" ) ) AND ( LIMIT-TO ( LANGUAGE , "English" ) OR
LIMIT-TO ( LANGUAGE , "Spanish" ) )
```

Note on the adaptation. Relative to the paediatric protocol (CRD42023472442), the “AND NOT adults” clause and the animal-model / non-human exclusions have been removed, the year limit has been extended to capture publications up to 1 June 2026, and a causal-inference / study-design block has been added so that Mendelian-randomisation, longitudinal-cohort, quasi-experimental (bariatric surgery, weight loss, GLP-1 receptor agonists) and epigenome-wide studies are retrieved alongside the observational and mechanistic literature.
